# Bridging the Physical Health Gap in Mental Health Settings: A Ward-Based Multidisciplinary Educational Intervention

**DOI:** 10.1192/bjo.2025.10412

**Published:** 2025-06-20

**Authors:** Andrea Nahum, Hannah Johnson, Harriet Agnew, Jessica Dorr, Dilisha Simkhada

**Affiliations:** 1 Guy’s and St Thomas’ NHS Foundation Trust, London, United Kingdom; 2 South London and Maudsley NHS Foundation Trust, London, United Kingdom; 3 South London and the Maudsley NHS Foundation Trust, London, United Kingdom

## Abstract

**Aims:** Adults with severe mental illness face a significantly higher risk of morbidity and premature death from preventable physical health conditions, with life expectancy reduced by 10–20 years compared with the general population. Early identification and management of physical health issues in mental health settings are crucial to reducing these disparities. We designed a Quality Improvement Project (QIP) focused on enhancing physical health outcomes through a ward-based multidisciplinary educational intervention on an acute male psychiatric unit in London.

**Methods:** We conducted an initial questionnaire to determine the MDT’s general knowledge level around physical health. We also surveyed the MDT on which teaching topics would be most relevant and interesting. The first audit cycle evaluated the MDT’s knowledge in physical health and baseline confidence on specific physical health topics. Our intervention involved a six-week, ward-based short course on physical health for mental health professionals taught by the ward’s resident doctors. A re-audit measured post-intervention improvements in topic specific confidence and knowledge.

**Results:** Fourteen members of the MDT completed the initial knowledge questionnaire, which, along with our survey, helped identify common gaps in knowledge and guided the selection of teaching topics for our intervention. The selected topics were: (1) Identifying and Escalating Abnormal Vital Signs, (2) Managing an Acutely Unwell Patient, (3) Commonly Used Psychiatric Drugs and Side Effects, (4) Clozapine, (5) ECGs, and (6) Medical Emergencies in the Psychiatric Setting. Before and after each teaching session MDT attendees were asked to complete a questionnaire assessing their knowledge and confidence on the given topic. On average, each session was attended by six MDT members. Baseline confidence in individual topics varied but improved following the intervention. Post-session knowledge scores were higher compared with pre-session assessments, demonstrating the effectiveness of the teaching sessions.

**Conclusion:** Ward-based interventions are effective in bridging gaps in physical health knowledge and improving confidence among MDT members. As learning is a continuous process, single teaching sessions may not be sufficient. On our ward, our QIP has led to the continuation of MDT teaching sessions by resident doctors. To further expand the impact of our QIP, we plan to extend MDT teaching to other wards within our Trust. To support this, course materials and summary infographics have been made available online, ensuring accessibility for our MDT and other wards looking to implement similar initiatives.

This work was carried out under the supervision of Dr Sarah Parry (Psychiatry Registrar) and Dr Jonathan Beckett (Psychiatry Consultant).

